# Measuring cerebrovascular reactivity with breath-hold fMRI in patients with Moyamoya angiopathy: MR perfusion based delay correction significantly improves agreement to [^15^O]water PET

**DOI:** 10.1007/s00234-025-03649-3

**Published:** 2025-05-24

**Authors:** Leonie Zerweck, Uwe Klose, Constantin Roder, Deborah Staber, Emely Renger, Ganna Blazhenets, Kathrin Grundmann-Hauser, Philipp T. Meyer, Ulrike Ernemann, Till-Karsten Hauser

**Affiliations:** 1 Department of Diagnostic and Interventional Neuroradiology, University Hospital Tuebingen, Tuebingen, Germany; 2 Department of Neurosurgery, University Hospital Tuebingen, Tuebingen, Germany; 3 Department of Nuclear Medicine, Medical Center– University of Freiburg, Faculty of Medicine, University of Freiburg, Freiburg, Germany; 4 Institute for Medical Genetics and Applied Genomics, University Hospital Tuebingen, Tuebingen, Germany

**Keywords:** Breath-hold fMRI, DCE MRI, [^15^O]water PET, Moyamoya angiopathy

## Abstract

**Purpose:**

Breath-hold functional MRI (bh-fMRI) is able to quantify cerebrovascular reactivity. Vessel stenoses can lead to delayed hemodynamic responses. We aimed to investigate whether delay correction improves the quality of bh-fMRI compared to the diagnostic standard [^15^O]water PET.

**Methods:**

The bh-fMRI data sets of 25 patients with Moyamoya Angiopathy were analyzed retrospectively without and with delay correction. Delay correction was calculated using time-to-peak (TTP) maps derived from dynamic susceptibility contrast (DSC) perfusion MRI. [^15^O]water PET maps and bh-fMRI maps without and with delay correction were presented blinded for delay correction to two neuroradiologists. The agreement between bh-fMRI without and with delay correction and [^15^O]water PET was independently and consensually rated on a 4-point-Likert scale (1 = poor, 2 = moderate, 3 = good, 4 = excellent) and compared with Wilcoxon signed-rank test.

**Results:**

The agreement between bh-fMRI and [^15^O]water PET without delay correction was good/excellent (median = 3, modus = 4), and improved significantly after delay correction with medium effect size (median = 4, modus = 4, z = -2.121, *p* = 0.034, *r* = 0.42).

**Conclusion:**

Delay correction improves the quality of bh-fMRI and seems to be helpful in clinical practice.

**Supplementary Information:**

The online version contains supplementary material available at 10.1007/s00234-025-03649-3.

## Introduction

Patients with Moyamoya Angiopathy (MMA) require hemodynamic evaluation to predict the risk of stroke and if necessary to indicate revascularization surgery [1–7]. Acetazolamide- (ACZ) triggered [^15^O]water PET is regarded as the diagnostic gold standard for assessing the cerebral perfusion reserve capacity (CPR) [8, 9]. The estimation of the cerebrovascular reactivity (CVR) by use of hypercapnia-triggered functional magnetic resonance imaging (fMRI) seems to be a more widely accessible alternative which yields results that are comparable to the diagnostic standard [1–3, 8]. The CVR is defined as the change in cerebral perfusion in response to vasodilatory stimulation, induced by breath-hold periods (bh-fMRI) [1–4, 10–12] or by the inhalation of CO_2_-enriched gas [8, 11–14]. Hypercapnia is a vasodilatory stimulus that evokes increased cerebral blood flow and thus a global Blood Oxygenation Level Dependent (BOLD) signal peak in healthy brain tissue [1, 10, 12]. In regions with reduced CVR, no or a reduced BOLD signal peak is expected [1, 5, 15]. Negative BOLD signals may even be observed in regions with so-called *steal phenomenon*, when perfusion in adjacent vascular territories increases at the expense of the severely affected region [1, 3, 12, 15–17].

It is known that the hemodynamic response can vary regionally [12, 18]. Vessel stenoses or blood flow via collateral vessels may result in delayed cerebral perfusion and lead to delayed BOLD signal peaks [18, 19]. This could lead to underestimated CVR values if time delays are not taken into account [19]. Therefore, recent studies discussed the need for delay correction in CVR assessment with hypercapnia-triggered fMRI [1, 12, 18, 19]. To the best of our knowledge, only one study compared bh-fMRI with and without voxel-wise delay correction to the established standard [^15^O]water PET [1]. In this study delays were estimated by calculating the optimal cross-correlation between each voxel’s time-course and a time-shifted cerebellar reference time-course. As result, a slightly improved agreement between bh-fMRI and [^15^O]water PET was observed [1]. It is worth mentioning that a limitation of this study was that the absolute temporal delays measured by MR perfusion were not considered.

Therefore, the aim of this study was to calculate the “real” time delays by use of dynamic susceptibility contrast (DSC) MR perfusion and to compare bh-fMRI with and without time delay correction to the diagnostic standard [^15^O]water PET in patients with MMA.

## Methods

### Study design

A retrospective analysis of bh-fMRI and the corresponding [^15^O]water PET data sets of patients with MMA was performed. The study was approved by the local ethics committee. Written informed consent for processing, archiving, and publication of their data was obtained from all participants.

### Patients

Inclusion criteria were angiographically diagnosed MMA and the availability of MRI (including bh-fMRI and DSC MRI) and [^15^O]water PET examinations a maximum of 4 months apart with no revascularization in between. Both examinations were performed as routine clinical scans. Exclusion criteria were other known cerebral diseases, acute stroke or cerebral hemorrhage. To avoid bias, a maximum of one data set (the first collected) of each patient was included.

### MRI data acquisition and processing

All MR images were acquired on a 3 T MR Scanner (Magnetom Skyra, Siemens, Erlangen, Germany) using a standard 20-channel head coil. A standardized MRI protocol was performed, including, among other sequences, a bh-fMRI and a DSC MRI sequence, as described more detailed below.

For DSC MRI, dynamic T2*-weighted images were acquired by using an echo-planar imaging (EPI) sequence with the following parameters: TR = 2340 ms, TE = 30 ms, matrix 128 × 128, slice thickness = 5 mm, 30 slices, FOV = 218 mm, resolution = 1.7 × 1.7 × 5.0 mm, TA = 1:58 min, 60 measurements. After 4 baseline measurements, a bolus of contrast agent (Gadobutrol, 0.15 mmol/kg of body weight) was administered intravenously at a rate of 3 ml/s, followed by a 25 ml saline flush at the same rate.

The fMRI data were acquired using T2*-weighted EPI sequences with the following parameters: TR = 3000 ms, TE = 36 ms, matrix 96 × 96, slice thickness = 3 mm, 34 slices in interleaved ascending order, FOV = 245 mm, resolution = 2.6 × 2.6 × 3.0, TA = 8:10 min, 181 measurements. The bh-task involved 60 s of normal breathing, followed by 7 repetitive cycles, each cycle consisting of 9 s end-expiratory breath-holding and 60 s of regular breathing. The respiratory instructions were presented visually via a wall-mounted display, utilizing a mirror affixed to the head coil. Scanner-triggered stimuli were presented using Presentation V20.1 (Neurobehavioral Systems, Berkeley, CA, USA).

Pre-processing of the bh-fMRI and DSC data was performed using Statistical Parameter Mapping (SPM12) running on MATLAB (R2018b (The MathWorks, Inc., Natick, MA). All DICOM images were converted to NIfTI (Neuroimaging Informatics Technology Initiative) format.

The DICOM images of the bh-fMRI series were slice-timing corrected, realigned and normalized to MNI space. Further data processing was conducted by using in-house scripts programmed in MATLAB [1–4, 10, 20, 21].

DSC MRI data were slice-timing corrected, realigned to correct head movement and normalized to standard Montreal Neuroimaging Institute (MNI) space. Voxel-wise Time-to-peak (TTP) maps were calculated and the prepocessed bh-fMRI data was corrected for bolus arrival time for each voxel.

The first step of the bh-fMRI CVR calculation was to verify patient compliance in performing the breath-hold task. As proposed by Hauser et al., the mean cerebellar time course of each of the 7 breath-hold periods was calculated [3]. The cerebellar time course is expected to reveal a physiological hemodynamic response in patients with MMA, as the cerebellum is supplied by vertebrobasilar arteries and MMA predominantly affects the territories of the internal carotid artery (ICA) [1–3, 6]. Periods with no expected cerebellar BOLD signal peak in visual inspection were excluded from the further analysis [3, 20]. All included breath-hold periods were averaged [3].

Each voxel’s signal time-course was detrended to remove linear signal changes over time. The percentage BOLD signal change relative to baseline was calculated.

The CVR was calculated for each of the 116 ROIs, both without (CVR _uncorrected_) and with delay correction (CVR _corrected_):

– To estimate the CVR _uncorrected_, the mean percentage BOLD signal change at the time point of the maximum cerebellar signal change ± 3 s was calculated.
– To calculate the CVR _corrected_, the DSC MRI data was used. The TTP of each voxel was determined and the time shift relative to the global mean TTP was calculated. Each voxel’s signal time-course was time-shifted by this value. After this delay correction, the mean percentage BOLD signal change at the time point of the maximum cerebellar signal change ± 3 s was calculated.

The CVR values were presented as color-coded overlays on normalized standard brains.

### [^15^O]water PET data acquisition and processing

PET scans were acquired as part of the clinical routine MMA evaluation on a Philips Vereos digital PET/CT system and analyzed as previously described [1, 2]. Summarized, two 4-min PET scans each (bolus injection of 291.2 ± 23.7 MBq [^15^O]water per scan) were performed before (baseline) and after a 5-min infusion (10 mL) of a standard dose of 1000 mg acetazolamide (ACZ) (post-ACZ). The inter-scan interval was 10 min, with the ACZ infusion being started immediately after the second baseline scan (the first post-ACZ scan started 10 min after start of the ACZ infusion). CPR maps were calculated as voxel-wise signal change [%] between the averaged baseline and post-ACZ maps (in analogy to [22]), which were derived by integrating the dynamic PET images over 60 s after the arrival of the tracer in the individual patient’s brain [23]. CRP maps were displayed in 32 transaxial planes covering the entire brain using a harmonized “rainbow” color scale. The color scale was thresholded symmetrically around zero (i.e., max/min set to ± [mean value + 2 standard deviations of CPR in cerebellar and PCA territories]) and customized such that the zero transition (i.e., watershed in the case of CBF steal) was marked by three black bins within the 256-bin color-scale.

### Data evaluation and statistical analysis

The two-way mixed-effects intraclass correlation coefficient (ICC) between the CVR _uncorrected_ and the CVR _corrected_ was determined to estimate the impact of the delay correction on the CVR values.

The agreement between the CVR _uncorrected_ maps/the CVR _corrected_ maps and the [^15^O]water PET maps was rated independently and in consensus by two neuroradiologists, using a 4-point Likert scale (1 = poor agreement, 2 = moderate agreement, 3 = good agreement, 4 = excellent agreement). The maps were presented in a blinded manner with regard to the CVR correction and in a randomized order.

Modus and median of all ratings were calculated, as well as the inter-rater agreement using quadratic weighted Cohen’s kappa κ_w_. The κ_w_ values were interpreted as follows: 0–0.20 = slight agreement; 0.21–0.40 = fair agreement; 0.41–0.60 = moderate agreement; 0.61–0.80 = substantial agreement and 0.81–1 = almost perfect agreement [24].

The Wilcoxon signed-rank test for paired data was used to compare the consensus ratings of the agreement between the [^15^O]water PET maps and the CVR _uncorrected_ and CVR _corrected_ maps. The effect size values *r* were interpreted as follows: *r* > 0.1 = small effect; *r* > 0.3 = medium effect; *r* > 0.5 = large effect; [25]. *p* values below 0.05 were considered significant.

## Results

In total, 26 data sets of 24 patients without prior surgery and 2 patients with prior unilateral superficial temporal artery (STA)-middle cerebral artery (MCA) bypass surgery fulfilled the inclusion criteria. The data set of one patient had to be excluded from the final analysis due to an atypical signal time-course, suggesting insufficient compliance. Overall, the quality of the remaining 25 data was good and only 5/174 (2.9%) of the periods had to be excluded due to inadequate compliance during the breath-holding paradigm. General patient data is presented in Table 1.

**Table 1:** General patient data

Number of patients of included data sets	25
Age (median, range)	39, 16–66
Female: Male ratio	1.6:1
Days between MRI and PET data acquisition (median, range)	59, 1–110
Data sets without revascularization surgery	23
Data sets after revascularization surgery	2 (unilateral STA-MCA in each case)^a^

^a^*STA* superficial temporal artery, *MCA* middle cerebral artery

### Impact of the delay correction on the CVR data

**Fig. 1:**
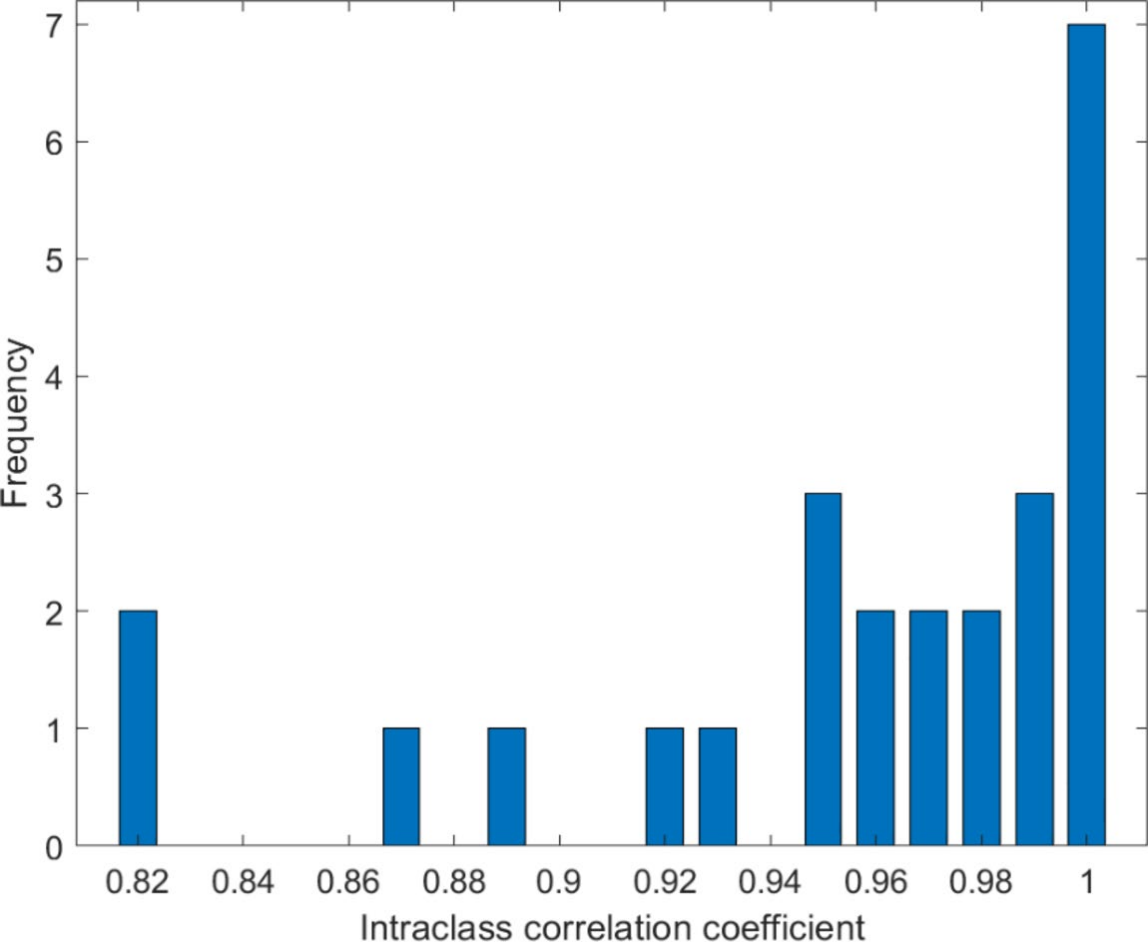
Histogram of the frequency distribution of the intraclass correlation coefficient between the cerebrovascular reactivity without (CVR _uncorrected_) and with time delay correction (CVR _corrected_) within the individual patients

The ICC of the CVR _uncorrected_ and the CVR _corrected_ within individual patients ranged from 0.82 to 1.00 (median = 0.97). The ICC of most patients was notably higher than 0.9 (see Fig. 1).

### Comparison of the CVR_uncorrected_ maps and the CVR_corrected_ maps with the [^15^O]water PET maps

Both raters independently rated a good to excellent agreement between the CVR _uncorrected_ maps and the [^15^O]water PET maps (rater 1: median = 3, modus = 4; rater 2: median = 3.5, modus = 4) (see Table 2 and supplementary Table 1A). The inter-rater reliability was almost perfect (κ_w_ = 0.79, 95% confidence interval (CI) = 0.62–0.97, *p* < 0.001). The consensual rating resulted in a good agreement (median = 3, modus = 4).

Evaluation of the agreement between the CVR _corrected_ maps and the [^15^O]water PET maps revealed an excellent agreement in the independent and in the consensual rating (median = 4, modus = 4 in all cases) (see Table 2 and supplementary Table 1B). The inter-rater reliability was substantial (κ_w_ = 0.69, 95% CI = 0.44–0.94, *p* < 0.001).

**Fig. 2:**
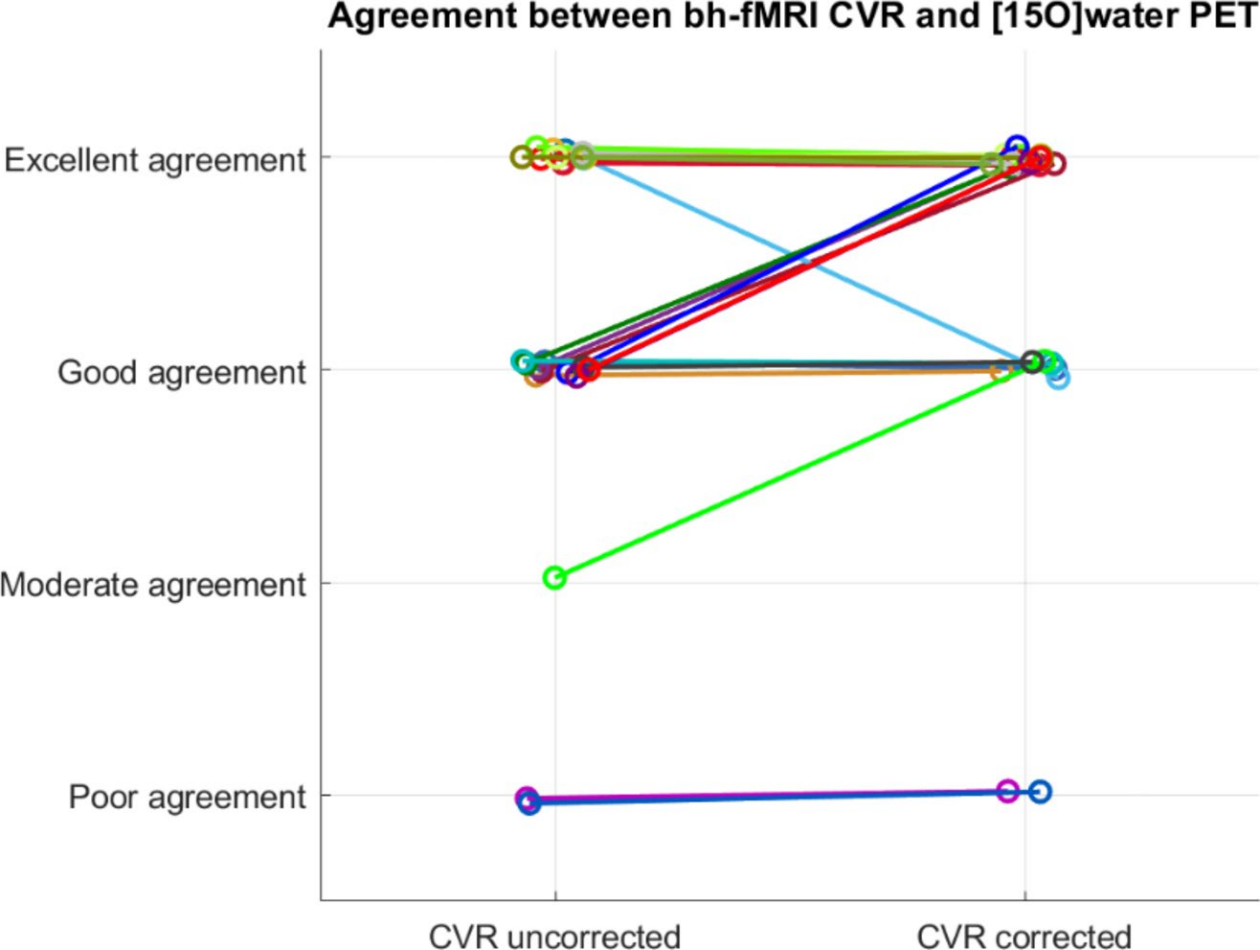
Consensus rating of the agreement between the CVR and the [^15^O]water PET maps without and with delay correction. Each color represents the data sets of one patient

The delay correction significantly improved the consensual agreement between the CVR and the PET maps (*z* = −2.121, *p* = 0.034, *r* = 0.42). Figure 2 shows that the rating remained the same or improved for most patients after delay correction and worsened for one patient.

**Table 2:** Consensus rating of the agreement between the CVR and the [^15^O]water PET maps before and after delay correction

	CVR _uncorrected_	CVR _corrected_
	Frequency (%)	Frequency (%)
Poor agreement	2 (8.0%)	2 (8.0%)
Moderate agreement	1 (4.0%)	0 (0.0%)
Good agreement	10 (40.0%)	6 (24.0%)
Excellent agreement	12 (48.0%)	17 (68.0%)

**Fig. 3:**
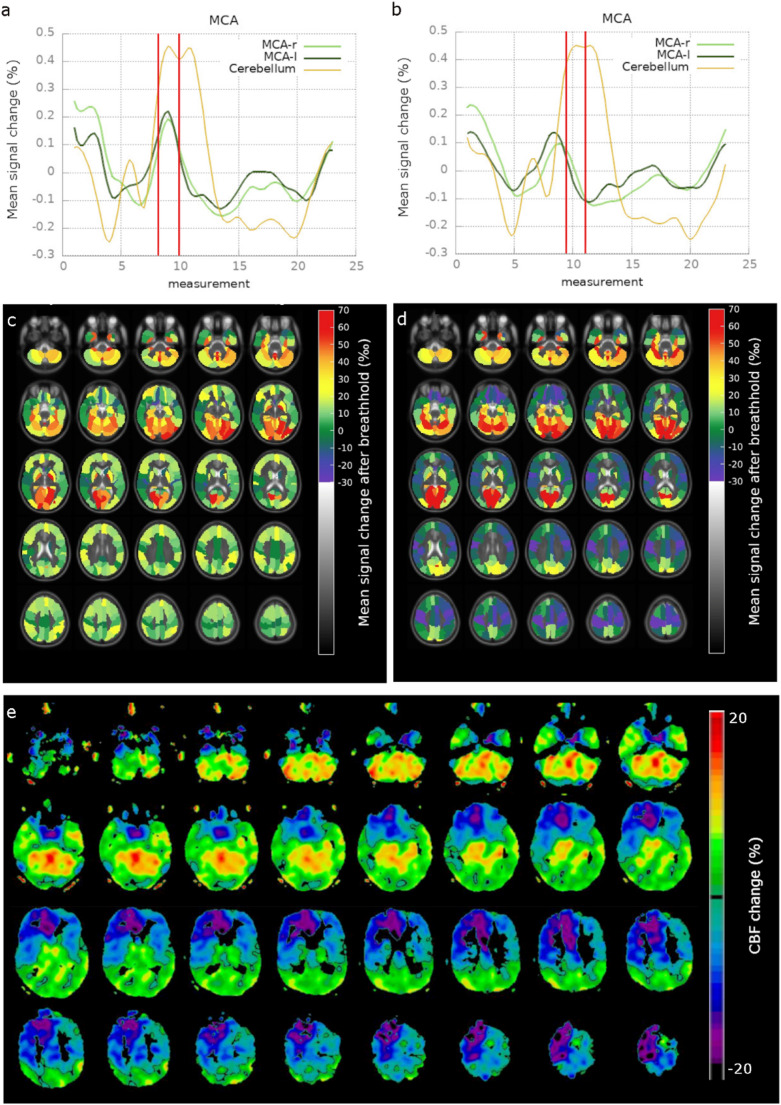
Breath-hold fMRI and [^15^O]water PET data of one patient who appears to benefit from delay correction. **a** and **b** show the mean signal time courses of the right and left middle cerebral artery (MCA-r, MCA-l) and of the cerebellum without (**a**) and with (**b**) delay correction. The red vertical lines show the time period of the maximum cerebellar signal change ± 3 s. Without delay correction, the maximum signal changes of the MCA-r and the MCA-l are smaller than the cerebellar maximum signal change but reach values > 0. The delay correction leads to time-shifted mean time courses of the MCA-r and the MCA-l, and results in even negative signal changes (steal phenomenon) at the time period of the maximum cerebellar signal change. The cerebrovascular reactivity (CVR) maps without (**c**) and with delay correction (**d**) and the corresponding [^15^O]water PET map (**e**) indicate that delay correction leads to a better agreement between breath-hold fMRI and [^15^O]water PET in this patient. The steal phenomenon in the territories of the MCA-r and the MCA-l, as observed in the [^15^O]water PET map, are more pronounced in the delay corrected CVR map than in the CVR map without delay correction

Exemplary data of one patient with improved agreement between the bh-fMRI CVR and the [^15^O]water PET maps after delay correction are shown in Fig. 3. The delay correction leads to a slightly different shape of the cerebellar signal peak and a different time point of the maximum cerebellar signal change. Without TTP correction the signal maxima of the right and left MCA are distinctly lower than the cerebellar signal maximum but reach their maximum at the same time point. The TTP correction results in shifts of the signal time courses of both right and left MCA, leading to negative signal changes at the time period of the maximum cerebellar signal change (steal phenomenon). Consecutively, the CVR _corrected_ maps were rated as more similar to the [^15^O]water PET maps than the CVR _uncorrected_ maps (consensus rating: Good agreement between CVR _uncorrected_ and [^15^O]water PET, excellent agreement between CVR _corrected_ and [^15^O]water PET). Since a bh-fMRI CVR impairment of > 50% is discussed as part of the indication for revascularization surgery [4], in this example the hemodynamic delay correction could lead to a different treatment decision based on the bh-fMRI data. The uncorrected bh-fMRI data demonstrate signal increases of about 45% of the cerebellar signal increase in both mean MCA territories, thereby rendering the surgical decision ambiguous. With TTP correction, the signal increases in both MCA territories are significantly reduced > 50%. According to the bh-fMRI data, this would clearly indicate a need for revascularization. However, these clear differences, which would in principle lead to a different recommendation of revascularization, were only visible in individual patients.

## Discussion

The aim of this study was to investigate whether hemodynamic delay correction based on DSC MRI impacts the diagnostic quality of bh-fMRI and improves the agreement with the gold standard [^15^O]water PET.

First, we calculated the ICC between the CVR _uncorrected_ and the CVR _corrected_ to estimate the impact of the delay correction on the bh-fMRI data. The high ICC values indicated that the delay correction resulted in slight changes to the bh-fMRI data.

Nevertheless, the CVR maps modified by time delay correction showed a significantly better agreement between the CVR maps and the [^15^O]water PET maps with a medium effect size. In most patients the delay correction resulted in the same or improved visual agreement between the bh-fMRI CVR maps and the [^15^O]water PET maps. However, it is important to note that in a small number of patients delay correction can reduce the level of agreement between bh-fMRI and [^15^O]water PET.

The small but significant differences in the bh-fMRI data after time delay correction, which in most cases resulted in improved data quality, need to be validated in a larger number of cases, but seem to justify the use of time delay correction in clinical routine. Further investigation with a large sample size is required to determine whether time delay correction of the bh-fMRI data has a significant impact on the indication for surgery, as suggested by individual cases in this study.

To the best of our knowledge, only one previous study has investigated the influence of delay correction on bh-fMRI in comparison to the gold standard [^15^O]water PET in patients with MMA [1]. Our findings are consistent with those of the aforementioned study, which reported a slight improvement of the delay corrected bh-fMRI data [1]. The authors did not acquire MR perfusion data; instead, they calculated the cross-correction between each voxel and the cerebellar reference time course to estimate the time delay of each voxel [1]. They warned that this method could reduce the agreement between bh-fMRI and [^15^O]water PET, particularly if a *recovery peak* following a steal phenomenon was incorrectly identified as a breath-hold induced BOLD signal peak [1]. By using perfusion MRI derived bolus arrival times, this study seems less prone to erroneous time shifting.

Most previous studies on hemodynamic delays in hypercapnia-triggered fMRI focused on estimating the time delay rather than acquiring MR perfusion data [12, 18, 22, 23, 26–28]. The delays were calculated using various techniques, including cross-correlation analysis between a voxel’s or region’s time course and a reference time course [12, 18, 26, 27]. Some studies also employed an iterative approach [18, 22] or used Fourier modeling [18, 23]. A key advantage of the method employed in this study is that MR perfusion data acquired during the same MR examination were used to calculate the *“real”* hemodynamic delay, as opposed to selecting a mathematical approximation. Previous studies that did not apply MR perfusion when calculating the hemodynamic delay reported higher CVR values after delay correction [19]. In contrast, this study demonstrated that CVR can also decline regionally when incorporating delay correction based on MR perfusion in the analysis, as illustrated by exemplary data from one patient.

This study has several limitations. One limitation of the study was the relatively small sample size, due to the low prevalence of MMA. Additionally, most patients included in the study had not undergone revascularization, making it unclear whether delay correction affects patients with extracranial-intracranial bypasses differently from those with indirect or no revascularization. Further prospective studies with larger sample size and a more heterogeneous patient population would help address this question. Another limitation was that the comparison between bh-fMRI and [^15^O]water PET maps relied on subjective ratings by two radiologists.

The method bh-fMRI has inherent limitations, including the dependence on patient compliance in performing the breath-hold paradigm [1–3, 29]. To address this limitation, we verified compliance during patient positioning in the scanner by checking breath-hold maneuvers personally and during data evaluation using a cerebellar signal time course-based method proposed by Hauser et al. which has been widely applied in other studies [1, 2, 4, 20]. In this study, we had to exclude the data from one patient due to poor compliance throughout the breath-hold paradigm. However, the compliance among the remaining patients was high, with only 2.9% of the remaining breath-hold periods needing exclusion. Another limitation is that, unlike hypercapnia-triggered fMRI with CO_2_ inhalation, bh-fMRI does not allow for absolute CVR quantification. This is due to breath-hold periods of the same duration produce varying BOLD signal changes across individual subjects [15, 29]. However, these limitations do not impact the method of this study, as the same data of a patient -with and without delay correction- were compared to their corresponding [^15^O]water PET data. Overall, this study reinforces the good agreement between bh-fMRI and the diagnostic standard [^15^O]water PET, MMA patients, consistent with previous studies [1, 3, 9].

## Conclusion

Delay correction based on MRI perfusion has been observed to have a modest impact on the bh-fMRI CVR. Nevertheless, it appears to significantly enhance the diagnostic accuracy and alignment with the gold standard [^15^O]water PET.

## Supplementary Information

Below is the link to the electronic supplementary material.

Supplementary file1 (DOCX 30 kb)

## Data Availability

No datasets were generated or analysed during the current study.
