# Supplementary material for: Measuring cerebrovascular reactivity with breath-hold fMRI in patients with Moyamoya angiopathy: MR perfusion based delay correction significantly improves agreement to [15O]water PET
Source: Neuroradiology. 2025 May 24;67(8):2121–9. doi: 10.1007/s00234-025-03649-3 (PMC12494653; doi:10.1007/s00234-025-03649-3)
Supplement: Supplementary file 1 — Supplementary file1 (DOCX 30 kb) [file 234_2025_3649_MOESM1_ESM.docx]

**Supplementary material**

**Supplementary Table 1A** Contingency table for the agreement between the CVR _uncorrected_ and the [^15^O]water PET maps presented in a blinded manner to raters 1 and 2

|  | | **Rater 1** | | | |
| --- | --- | --- | --- | --- | --- |
|  |  | **Poor agreement** | **Moderate**  **agreement** | **Good agreement** | **Excellent agreement** |
| **Rater 2** | **Poor agreement** | 2 | 0 | 0 | 0 |
|  | **Moderate agreement** | 0 | 0 | 2 | 0 |
|  | **Good agreement** | 0 | 1 | 7 | 2 |
|  | **Excellent agreement** | 0 | 0 | 0 | 11 |

**Supplementary Table 1B** Contingency table for the agreement between the CVR _corrected_ and the [^15^O]water PET maps presented in a blinded manner to raters 1 and 2

|  | | **Rater 1** | | | |
| --- | --- | --- | --- | --- | --- |
|  |  | **Poor agreement** | **Moderate agreement** | **Good agreement** | **Excellent agreement** |
| **Rater 2** | **Poor agreement** | 2 | 0 | 0 | 0 |
|  | **Moderate agreement** | 0 | 0 | 1 | 0 |
|  | **Good agreement** | 0 | 0 | 4 | 2 |
|  | **Excellent agreement** | 0 | 0 | 4 | 12 |
